# Supplementary material for: Prevalence and Predictors of the Use of Low-Calorie Sweeteners Among Non-Pregnant, Non-Lactating Women of Reproductive Age in Australia
Source: Nutrients. 2024 Nov 20;16(22):3963. doi: 10.3390/nu16223963 (PMC11597300; doi:10.3390/nu16223963)
Supplement: Supplementary file 1 [file nutrients-16-03963-s001.zip › nutrients-3252286-supplementary.pdf]

Supplementary Material

Supplementray Table S1: Akaike's information criterion and Bayesian information criterion

Model	N	AIC	BIC	R2
One class	405	5598.971	5667.037	
Two class	405	5276.361	5416.497	0.78
Three class	405	5124.758	5336.964	0.83
Four class	405	5446.661	5726.933	0.85
Five class	405	5051.027	5363.331	0.83

Based on lowest BIC and AIC, we chose 3 class model

Supplementary Figure S1 : Estimated response probability of low-calorie sweetened food and drink consumption in each latent class

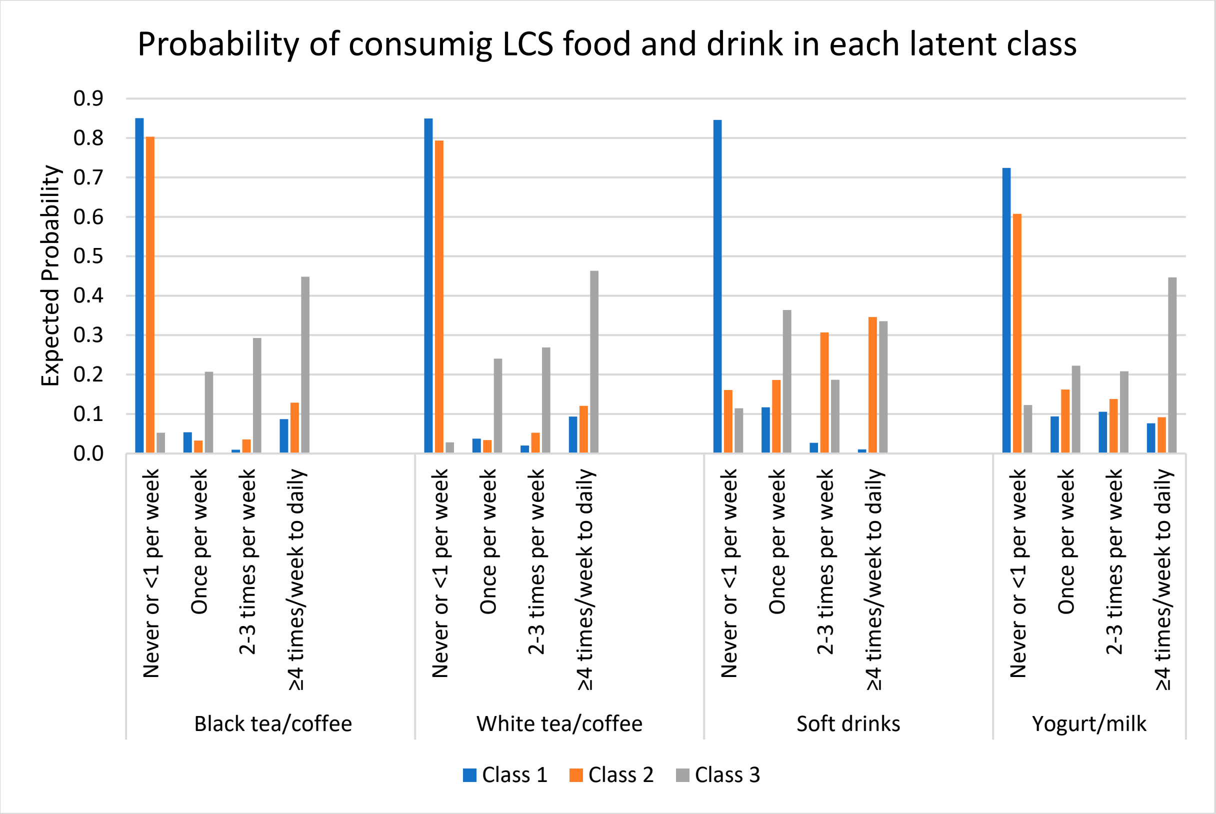

Supplementary Figure S2: Probability of regularly using LCS food and drink in each latent class

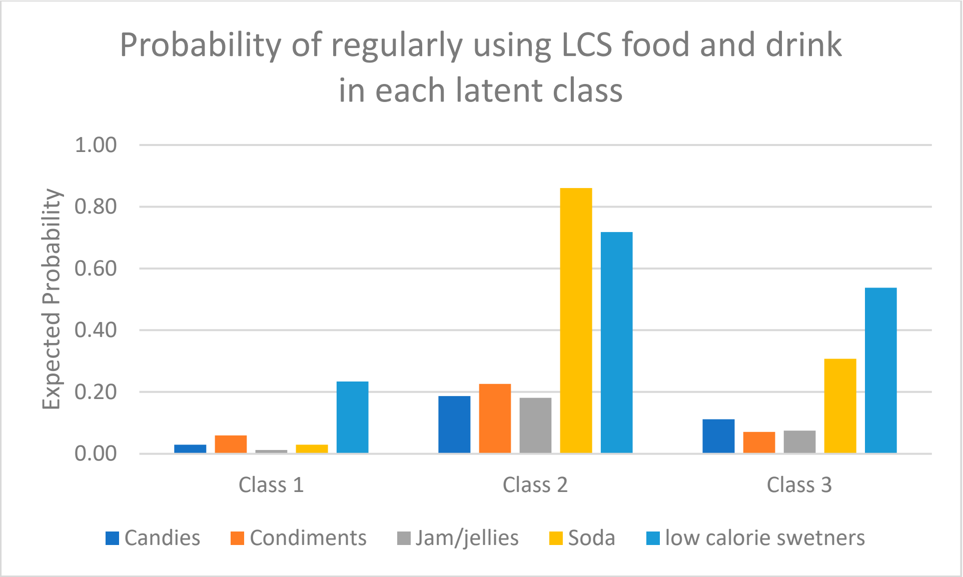
Probability of regularly using LCS food and drink in each latent class
Expected Probability
1.00
0.80
0.60
0.40
0.20
0.00
Class 1
Class 2
Class 3
Candies
Condiments
Jam/jellies
Soda
low calorie swetners
